# A hierarchical optimization approach to maximize hosting capacity for electric vehicles and renewable energy sources through demand response and transmission expansion planning

**DOI:** 10.1038/s41598-024-66688-5

**Published:** 2024-07-09

**Authors:** Sulaiman Z. Almutairi, Abdullah M. Alharbi, Ziad M. Ali, Mohamed M. Refaat, Shady H. E. Abdel Aleem

**Affiliations:** 1 Department of Electrical Engineering, College of Engineering, Prince Sattam Bin Abdulaziz University, 16278 Al Kharj, Saudi Arabia; 2 Department of Electrical Engineering, College of Engineering at Wadi Addawasir, Prince Sattam Bin Abdulaziz University, Wadi Addawasir, Saudi Arabia; 3 Department of Electrical Engineering, Aswan Faculty of Engineering, Aswan University, Aswan, 81542 Egypt; 4 Department of Photovoltaic Cells, Electronics Research Institute, Cairo, 11843 Egypt; 5 Department of Electrical Engineering, Institute of Aviation Engineering and Technology, Giza, 12658 Egypt

**Keywords:** Demand response, Electric vehicles, Hosting capacity, Renewable energy sources, Transmission expansion planning, Electrical and electronic engineering, Energy grids and networks

## Abstract

Within the scope of sustainable development, integrating electric vehicles (EVs) and renewable energy sources (RESs) into power grids offers a number of benefits. These include reducing greenhouse gas emissions, diversifying energy sources, and promoting the use of green energy. Although the literature on hosting capacity (HC) models has grown, there is still a noticeable gap in the discussion of models that successfully handle transmission expansion planning (TEP), demand response (DR), and HC objectives simultaneously. Combining TEP, DR, and HC objectives in one model optimizes resource use, enhances grid stability, supports renewable and EV integration, and aligns with regulatory and market demands, resulting in a more efficient, reliable, and sustainable power system. This research presents an innovative two-layer HC model, including considerations for TEP and DR. The model determines the highest degree of load shifting appropriate for incorporation into power networks in the first layer. Meanwhile, the second layer focuses on augmenting the RES and EVs’ hosting capability and modernizing the network infrastructure. System operators can choose the best scenario to increase the penetration level of EVs and RESs with the aid of the proposed model. The proposed model, which is formulated as a multi-objective mixed-integer nonlinear optimization problem, uses a hierarchical optimization technique to identify effective solutions by combining the particle swarm optimization algorithm and the crayfish optimizer. When compared to traditional methods, the results obtained from implementing the proposed hierarchical optimization algorithm on the Garver network and the IEEE 24-bus system indicated how effective it is at solving the presented HC model. The case studies demonstrated that integrating DR into the HC problem reduced peak load by 10.4–23.25%. The findings also highlighted that DR did not impact the total energy consumed by EVs throughout the day, but it did reshape the timing of EV charging, creating more opportunities for integration during periods of high demand. Implementing DR reduced the number of projects needed and, in some cases, led to cost savings of up to 12.3%.

## Introduction

There is currently a greater need for environmental sustainability due to global imperatives. In order to counteract climate change and reduce greenhouse gas emissions, the widespread adoption of renewable energy sources (RESs) is paramount^1,2^. Notably, the proliferation of electric vehicles (EVs) has witnessed significant growth in recent years^3^. The integration of EVs into power systems not only signifies a crucial development but also provides a distinctive opportunity to strengthen grid flexibility, facilitate the continuous integration of renewable energy resources, optimize demand response (DR) mechanisms, and contribute substantially to the creation of a more sustainable and efficient energy system^4–6^.

### Background motivation

Improving the hosting capacity (HC) of EVs and RESs requires strategic planning, enacting regulations that support them, and efficient stakeholder cooperation. This comprehensive strategy is necessary to optimize the advantages and guarantee a smooth transition in the changing energy environment^7–9^. Power networks face significant issues due to the growing need for EV charging and the widespread use of RESs. Many of the current systems are not equipped to handle the expected increase in demand^10,11^. In practical terms, a noticeable conflict of interest has emerged between owners and investors of distributed generation (DG) and distribution system operators. Although DG investors support greater incorporation of DG into power networks, system operators have reasonable worries about possible problems associated with excessive DG penetration^12–14^. These concerns span a spectrum of operational limit violations, encompassing over and under voltages, excessive line losses, overloading of transformers and feeders, protection failure, and elevated levels of harmonic distortion that surpass international standards. These issues manifest when the system surpasses its HC threshold^15–17^. To successfully navigate the intricacies of this dynamic energy landscape, a careful balance between the interests of stakeholders and the preservation of system integrity is essential.

### Literature review

Increasing HC is a difficult task that calls for a combination of behavioral, governmental, and technical initiatives^18^. The synergistic implementation of these strategies is pivotal for steering power systems toward sustainability and resilience, particularly in accommodating the escalating presence of clean energy sources and EVs^19,20^. Transmission expansion planning (TEP) is a cornerstone in this pursuit, recognized for its indispensable role in fortifying the HC of RESs and EVs^21^. TEP involves strategically evaluating and expanding the existing transmission infrastructure to adeptly meet current and projected electricity demands. Its impact extends beyond enhancing grid resilience and flexibility by modernizing transmission infrastructure, facilitating the integration of intermittent RESs such as wind and solar, and addressing the increasing demand for EVs^22^. Additionally, TEP catalyzes incorporating remote renewable resources into the grid, particularly in distant areas. Its significance is underscored in electrifying transportation, ensuring reliable electricity delivery for EV charging, mitigating grid congestion issues, and facilitating the efficient distribution of renewable energy^23,24^. TEP emerges as an indispensable enabler for the integration of clean energy into energy markets, thereby fostering economic viability and competitiveness for RESs^25^.

A comprehensive multi-stage TEP framework, encompassing both investment and operation aspects related to Grid-to-Vehicle (G2V), Vehicle-to-Grid (V2G), and Vehicle-to-Building (V2B) systems, was proposed in Borozan et al.^26^. Case studies conducted within this framework illustrated its efficacy in determining strategic measures. This innovative approach facilitates informed decision-making concerning optimal locations, capacities, and timing for smart charging investments, thereby enabling the deferral or replacement of traditional infrastructure reinforcements. Addressing voltage stability challenges arising from high EV and RES penetration, a novel TEP approach was introduced by Abdi et al.^27^. Results indicated that integrating reactive power planning with TEP enhances power system reliability with minimal additional investment. The authors in Mirzaei et al.^28^ presented a practical strategy for the long-term expansion planning of EV parking facilities, focusing on maximizing operator profits. Comparative outcomes revealed energy savings through the proposed parking selection approach. Authors in Tao et al.^29^ introduced an advanced planning model encompassing multiple stages, considering distribution network assets and EV charging stations. The evaluation of service quality metrics, including average waiting time and the average number of unsatisfied customers, ensured the provision of high-quality fast-charging services as EV adoption increased. The authors in Gbadamosi et al.^30^ addressed power quality enhancement through TEP, allowing extensive RES integration while adhering to harmonic emissions constraints. The authors in^31^ introduced an innovative planning model focusing on carbon emission reduction in integrated systems with EV fast-charging stations. Case study findings emphasized the significant influence of carbon emission constraints on planning outcomes, revealing the intricate relationship between infrastructure installation in EV charging stations and integrated natural gas systems based on specified carbon emission limits.

Demand Response (DR) is a pivotal tool in optimizing energy consumption, fortifying grid reliability, and bolstering the HC of RESs and EVs, benefiting consumers, grid operators, and the environment^32,33^. Its integration into TEP provides a flexible and cost-effective strategy to meet future electricity demand, leveraging existing infrastructure, deferring costly upgrades, and enhancing grid efficiency^34^. Notably, DR can potentially mitigate the necessity for transmission upgrades by managing demand during peak periods. However, transmission planners need to carefully assess how cost-effective DR is in comparison to more conventional projects^35^. In Hamidpour et al.^36^, a comprehensive approach to power system expansion planning was presented, incorporating local wind farms, energy storage systems (ESSs), and incentive-driven DR initiatives. Results underscored the effectiveness of DR in reducing overall planning costs. The authors in Davoodi et al.^37^ introduced a multifaceted framework for power system expansion planning, emphasizing the integration of DGs and the implementation of load management and DR algorithms, introducing a new dimension to the planning process.

HC models typically fall into two categories: robust HC models and stochastic HC models^38,39^. Robust models consider a spectrum of scenarios for integrating RES and EVs, striving to identify the optimal expansion plan accommodating all possibilities. Here, the HC model calculates the maximum allowable capacity for RES or EV under diverse grid conditions. Conversely, stochastic models inject uncertainty into the planning process, incorporating probabilistic scenarios for RES generation and EV adoption. Stochastic HC models in TEP assess the probability of exceeding grid limits, aiming to maximize RES and EV integration while upholding reliability. In Alnowibet et al.^40^, a TEP model employing robust optimization was introduced for determining the optimal configuration of a transmission grid. Unlike conventional approaches with a single uncertainty set, this model advocated incorporating multiple uncertainty sets, each with its associated probability. The authors in Bødal et al.^41^ proposed an approach to integrate stochastic power system operations into day-ahead and balancing electricity markets, utilizing a power expansion model. Employing bender cuts and a stochastic rolling horizon dispatch, the model considered various cut formulations to model operational expenses in the expansion problem, revealing that cuts derived from the day-ahead problem yielded the lowest expected total cost. In Sani et al.^42^, the authors introduced a robust methodology representing the interaction between a substantial fleet of EV users and utility companies within the context of long-term generation expansion planning. Findings highlighted that a generation expansion plan, robust and behaviorally consistent, could lower the actual total cost of the system compared to a plan lacking such behavioral consistency.

Determining the HC model is crucial since it presents a nonlinear optimization problem^43,44^. Traditionally, mathematical and metaheuristic optimization algorithms are applied to tackle the HC problem. Mathematical programming optimization algorithms are robust tools for discovering optimal solutions across diverse applications^45,46^. However, they grapple with challenges related to computational complexity, model assumptions, and sensitivity to problem formulation changes, especially in dealing with large-scale or intricate nonlinear problems^47^. Non-convex optimization problems, common in HC models, may have multiple local optima, complicating the guarantee of global optimality by mathematical algorithms^48^. Metaheuristic algorithms, while offering flexibility and efficiency in solving complex optimization problems, come with the trade-off of providing approximate solutions that may not consistently meet desired quality standards^49^. As heuristics rather than optimization algorithms, metaheuristics do not always guarantee finding the global optimum, and their effectiveness often hinges on the fine-tuning of numerous parameters, a process that can be time-consuming^50^.

### Research gaps and contributions

The discussion above highlights the benefits of DR programs in facilitating the incorporation of RES and EVs, as well as the significance of TEP in modernizing power networks to meet future energy demand. By integrating TEP, DR, and HC objectives into a unified model, it becomes feasible to enhance resource utilization, bolster grid stability, and facilitate the integration of renewable energy sources and EVs. This comprehensive approach not only reduces costs and ensures a dependable energy supply but also aligns with regulatory requirements. Furthermore, it fosters sustainability, promotes effective energy utilization, and prepares the grid to address forthcoming challenges.

**Figure 1:**
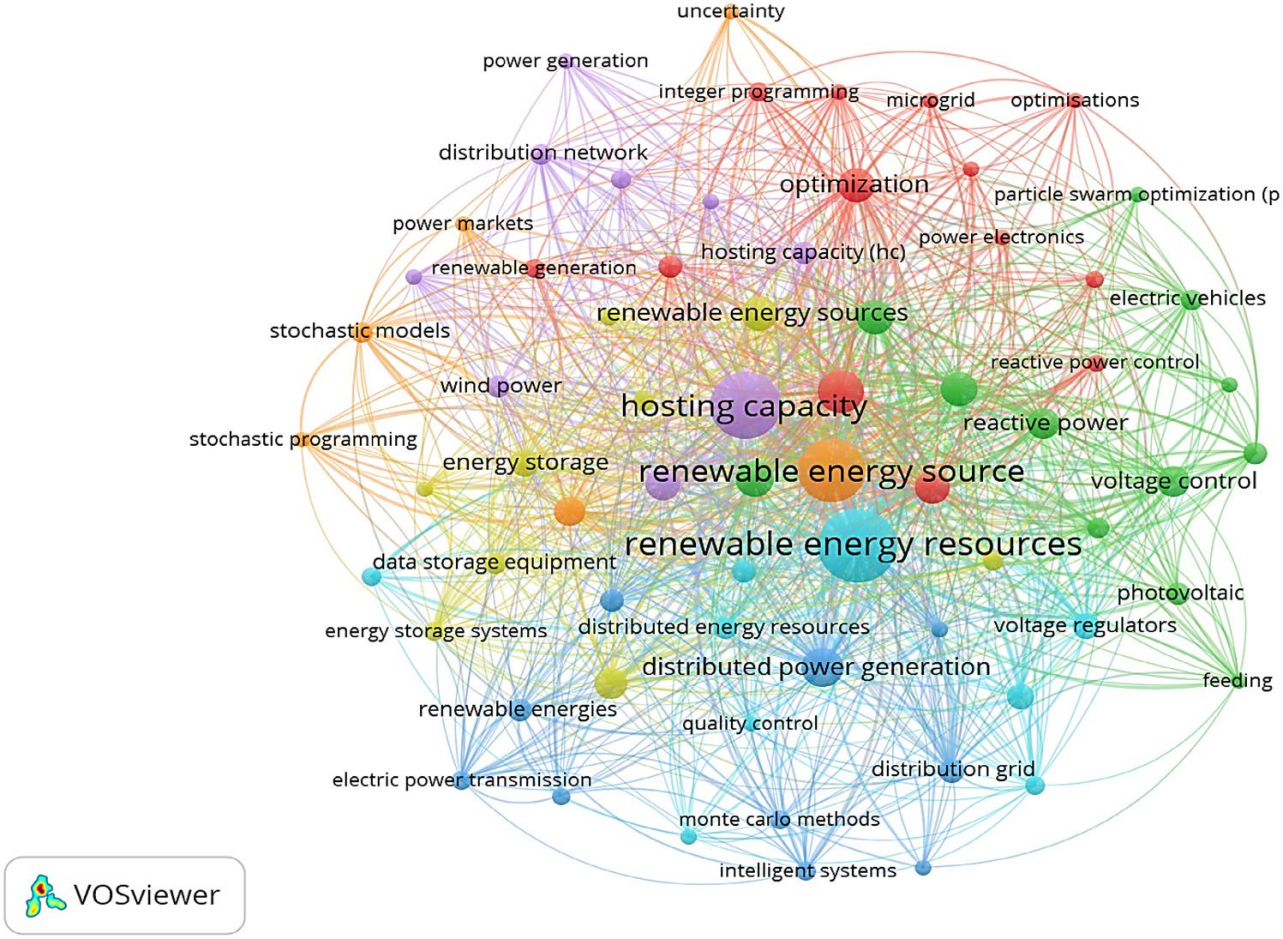
Co-occurrence analysis map of articles focusing on HC.

A considerable portion of HC models has been presented in the existing literature. However, there is a notable gap in discussions focusing on models that concurrently address HC targets, DR, and TEP, as depicted in Fig. 1. Most research tends to integrate either TEP or DR with EV and RES HC models, often neglecting the untapped benefits of integrating both models simultaneously for an enhanced HC framework.

In this work, the hybrid HC planning model is presented. It considers TEP for network upgrading, DR, and the HC estimation of EV and RES. In addition, a hierarchical optimization scheme is developed to simplify the problem. To guarantee high-quality solutions, a hybrid meta-heuristic algorithm is developed.

**Figure 2:**
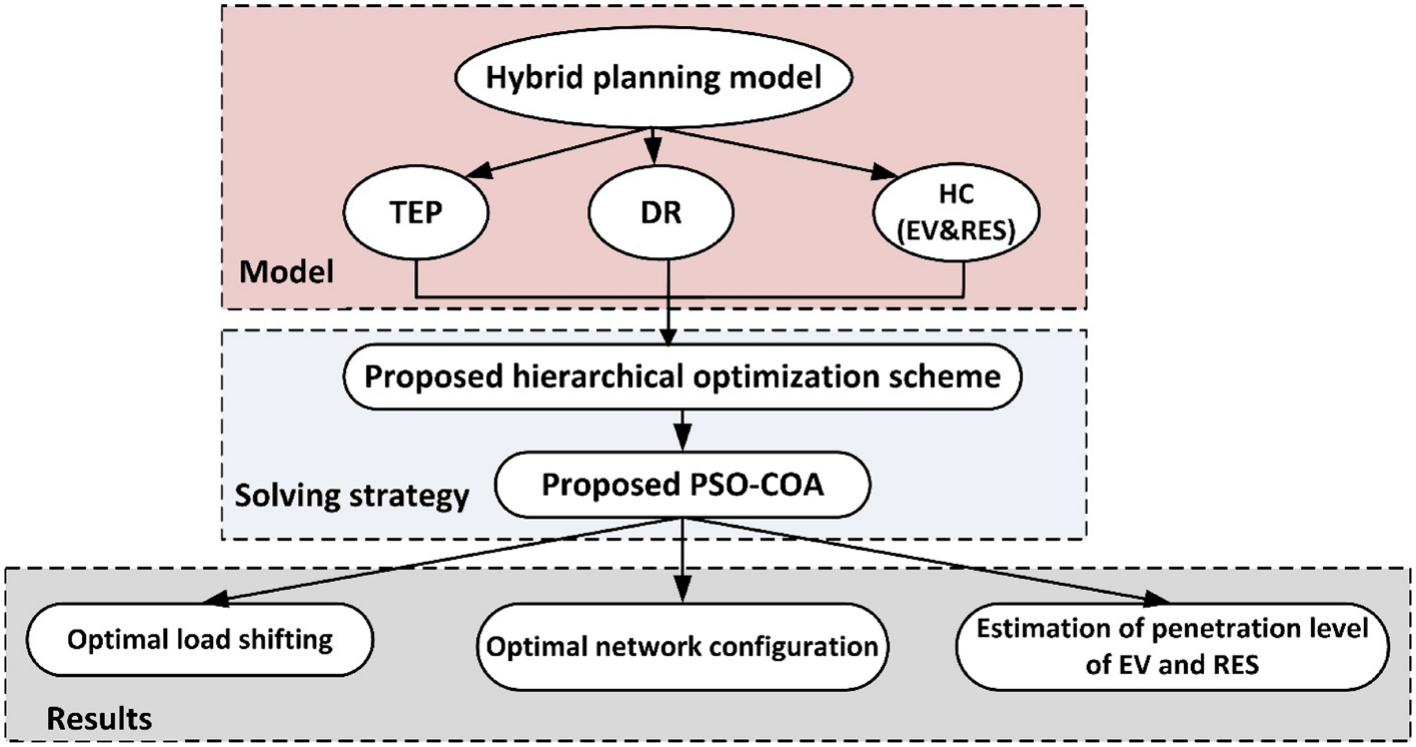
Proposed HC planning scheme.

The proposed planning strategy is illustrated in Fig. 2. The proposed framework begins by determining the network's maximum load-shifting capacity to ensure system reliability. This sets the load-shedding value, which controls the level of EV penetration to keep the total load within acceptable limits. The second phase focuses on increasing the penetration of RES and EVs without compromising network security. The final phase involves upgrading infrastructure and optimizing the network configuration to support the expected increase in RES and EV usage.

Table 1 highlights the main contributions of the current work compared to some studies in the literature. This paper’s primary contributions can be briefly summarized as follows:

- The development of an HC model is effectively included in DR and TEP. The final capacity of power networks to support RES and EVs is determined by this model, which synergizes between DR and TEP.
- The proposed model seeks to facilitate the integration of various components, including static var compensators (SVCs), ESSs, and fault current limiters (FCLs), enhancing HC technically and economically.
- A hierarchical optimization algorithm is formulated to solve the proposed model, decomposing the planning model into several levels for problem simplification and generating high-quality solutions.
- Introduction of a novel hybrid multi-objective optimization algorithm based on the crayfish optimization algorithm and the particle swarm optimizer, named PSO-COA, specifically tailored to solve the suggested model, contributing to the advancement of methodologies in this domain.

**Table 1:** Highlights of the main contributions of the current work and some studies in the literature.

References	Year	Considered models	Solved strategy
Borozan et al.^26^	2021	Multi-stage TEP considering EV demand, RES, and network reinforcement	A hierarchical multi-cut Benders decomposition was applied
Abdi et al.^27^	2021	The hybrid model of reactive power planning and TEP considers EV and RES	The imperialist competitive algorithm was recommended
Mirzaei et al.^28^	2022	The long-term EV expansion planning	The non-dominated sorting GA was applied
Gbadamosi et al.^30^	2022	TEP model that considers RES and power harmonics	CPLEX and XA solvers were used
Wu et al.^31^	2022	Planning model that considers natural gas systems, RES, and EV	GA was applied
Davoodi et al.^37^	2022	TEP with RES	The adaptive PSO was used
Alnowibet et al.^40^	2022	Robust TEP model	The classical column-and-constraint generation technique was applied
Bødal et al.^41^	2022	The expansion model considers a rolling horizon framework	The bender decomposition technique was used
Current work	–	A hybrid TEP model that considers DR and the concept of EV and RES hosting capacity	A hierarchical optimization scheme is proposed to simplify the problem. In addition, the hybrid PSO-COA is developed

### Organization

The remaining sections of the paper are organized as follows: the methodology is presented in Sect. “Methodology”, while the proposed solving strategy and PSO-COA algorithm are introduced in Sect. “Optimization algorithm”. Section “Results and discussion” is dedicated to presenting the results and discussions, and the work is concluded in Sect."Conclusions".

## Methodology

**Figure 3:**
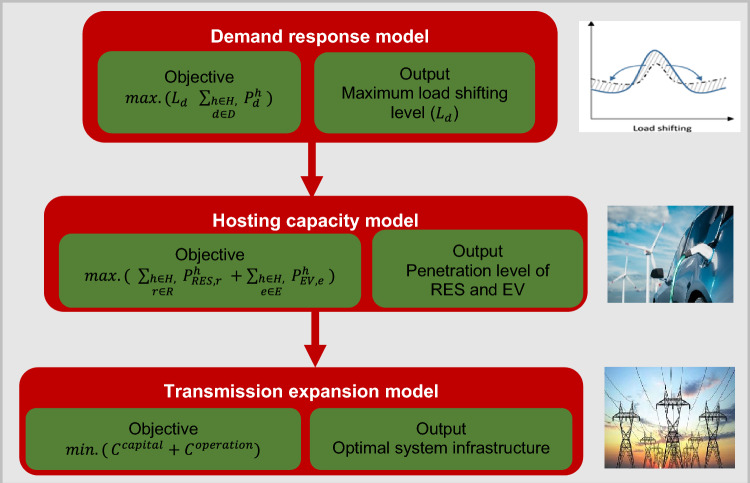
Proposed HC model.

A visual representation of the proposed framework for integrating HC, TEP, and DR is depicted in Fig. 3. The proposed model comprises three levels. In the first level, the DR program is used to refine the definition of the maximum load-shifting level to enhance the penetration of renewables and EVs without compromising system reliability. The second level incorporates the HC model to maximize the integration levels of RES and EVs, considering both economic and technical aspects provided in the third level. The third tier entails infrastructure enhancement, determining the optimal configuration of transmission lines, ESSs, FCLs, and SVCs to accommodate the anticipated increase in RES and EV usage.

### Demand response model

DR programs are prevalent in adjusting electricity consumption, either by curtailing or shifting usage during specific periods. This adjustment is often influenced by factors such as electricity prices, financial incentives provided by system operators, or emergencies requiring immediate balancing of supply and demand for system stability. The proposed DR model focuses on identifying the maximum achievable level of load demand shifting (*L*_*d*_) that the network can accommodate. This strategic shift aims to align with daily load energy requirements while maintaining system reliability and stability.

The considered DR model is formulated as an optimization problem to maximize the load shifting level, as shown in (1):

1 $${OF}_{1}=max. ({L}_{d} \sum_{h\in H}\sum_{i\in D}{ P}_{d,i}^{h} )$$

The model constraints elaborate on guaranteeing that the load energy requirement over the day is at least met at the end of the day, as described in (2) and (3).

2 $$\sum_{h\in H}\sum_{i\in D}\left(1-{L}_{d}\right) \overline{{P }_{d,i}^{h}} \Delta h=\sum_{i\in D}{E}_{d,i} ; \forall \left\{h\in H,i\in D \right\}$$

3 $$\sum_{i\in D}\left(1-{L}_{d}\right) \overline{{P }_{d,i}^{h}} \le { P}_{d}^{max} ; \forall \left\{i\in D \right\}$$

where,

4 $$\overline{{P }_{d,i}^{h}}={ P}_{d,i}^{h}+\sum_{d\in D}\sum_{t\in T}\overline{{P }_{d,i}^{t}} {L}_{d} ; \forall \left\{t\in T,i\in D\right\}$$

The symbol ${E}_{d,i}$ signifies the overall energy demand at bus *i* required to be supplied throughout the day. ${P}_{d,i}^{h}$ represents the real load demand at hour *h* for bus *i*. The notation ${P}_{d,i}^{h}$ denotes the load demand at hour *h* for bus *i* resulting from applied load shifting, as outlined in (4). This includes both the actual load demand of bus *i* and the load shifted in previous hours due to surpassing the maximum permissible limit. *H* and *D* denote sets of representative hours and load nodes, respectively. It is crucial to emphasize that the total load at any given hour should not exceed the predefined value (${P}_{d}^{max}$​), expressed in (3).

### HC model

The fundamental aim of the model is to optimize the integration levels of RES and EV while upholding system reliability. The objective function can be succinctly expressed as follows:

5 $${OF}_{2}=max. ( \sum_{\begin{array}{c}h\in H,\\ r\in R\end{array}}{ P}_{RES,r}^{h} +\sum_{\begin{array}{c}h\in H,\\ e\in E\end{array}}{ P}_{EV,e}^{h} )$$

where ${P}_{RES,r}^{h}$ represents the generated power of RES at bus *r* in MW and ${P}_{EV,e}^{h}$ denotes the EV charging load in MW at bus *e*. *R* and *E* indicate sets of RES and EV charging station locations.

The HC model adheres to specific constraints to uphold network reliability and stability. Equations (6) and (7) delineate the restrictions for EV and RES. Equations (8) and (9) evaluate the active and reactive power capacities of conventional generation units, represented by ${P}_{T,g}^{h}$ and ${Q}_{T,g}^{h}$, respectively. As demonstrated in (6), the EV power level (${P}_{EV,e}^{h}$) is bounded by the load shifted at any given hour and the permissible increase above the peak load ($\overline{{\Delta P }_{EV}}$). To prevent surpassing generation station capacities, both renewable and conventional units must adhere to specified limits for power output, ensuring compliance in steady-state and congestion scenarios. $\overline{{P }_{RES,g}^{h}}$ establishes the maximum output power limit of RES at hour *h*. $\underset{\_}{{P}_{T,g}^{h}}$ and $\overline{{P }_{T,g}^{h}}$ indicate the minimum and maximum active power injectable from conventional generation units while $\underset{\_}{{Q}_{T,g}^{h}}$ and $\overline{{Q }_{T,g}^{h}}$ refer to their minimum and maximum. *G* is a set containing the locations of conventional generation units.

6 $$0\le { P}_{EV,e}^{h}\le {L}_{d} \overline{{P }_{d}^{h}}+ \overline{{\Delta P }_{EV}}; \forall \left\{h\in H,e\in E\right\}$$

7 $$0\le {P}_{RES,r}^{h}\le \overline{{P }_{RES,r}^{h}}; \forall \left\{h\in H,r\in R\right\}$$

8 $$\underset{\_}{{P}_{T,g}^{h}}\le {P}_{T,g}^{h}\le \overline{{P }_{T,g}^{h}} ; \forall \left\{h\in H,g\in G\right\}$$

9 $$\underset{\_}{{Q}_{T,g}^{h}}\le {Q}_{T,g}^{h}\le \overline{{Q }_{T,g}^{h}} ; \forall \left\{h\in H,g\in G\right\}$$

The control of load bus angles (${\theta }_{i}^{h}$) and voltages (${V}_{i}^{h}$) at hour *h* is accomplished through (10) and (11), and it is essential to ensure that they remain within predefined bounds. $\underset{\_}{{\theta }_{i}^{h}}$ and $\overline{{\theta }_{i}^{h}}$ denote the lower and upper limits of the bus angles, while $\underset{\_}{{V}_{i}^{h}}$ and $\overline{{V }_{i}^{h}}$ represent the lower and upper limits of bus voltages. *B* represents a set of the system buses.

10 $$\underset{\_}{{\theta }_{i}^{h}}\le {\theta }_{i}^{h}\le \overline{{\theta }_{i}^{h}} ; \forall \left\{h\in H,i\in B\right\}$$

11 $$\underset{\_}{{V}_{i}^{h}}\le {V}_{i}^{h}\le \overline{{V }_{i}^{h}} ; \forall \left\{h\in H,i\in B\right\}$$

The load flow balance equations often represent the equality constraints, which can be expressed by (12) and (13). Meanwhile, the active and reactive powers transmitted through each circuit denoted as ${{P}_{ij}}^{h}$ and ${{Q}_{ij}}^{h}$, are governed by (14). ${G}_{ij}$ and ${B}_{ij}$ represent the conductance and susceptance of transmission lines, respectively. ${P}_{i}^{loss,h}$ and ${Q}_{i}^{loss,h}$ denote the active and reactive power losses through transmission circuits connected to bus *i*. ${P}_{ESS,b}^{ h}$ and ${Q}_{ESS,b}^{ h}$ refer to the active and reactive power shared by ESS at bus *i*.

12 $$\sum_{g\in {M}^{i}}{ P}_{g,i}^{h}-\sum_{b\in {M}^{i}}{P}_{ESS,b}^{ h}-0.5 \sum_{l\in {M}^{i}}{P}_{i}^{loss,h}+{{V}_{i}^{h}}^{2}{G}_{ij}-{V}_{i}^{h}{V}_{j}^{h} {G}_{ij}\mathit{cos}\left({\theta }_{i}^{h}-{\theta }_{j}^{h}\right)-{V}_{i}^{h}{V}_{j}^{h} {B}_{ij}\mathit{sin}\left({\theta }_{i}^{h}-{\theta }_{j}^{h}\right)={P}_{i}^{d,h} ; \forall \left\{h\in H\right\}$$

13 $$ \begin{aligned} & \sum_{g\in {M}^{i}}{ Q}_{T,g}^{h}-\sum_{b\in {M}^{i}}{Q}_{ESS,b}^{ h}-0.5 \sum_{g\in {M}^{i}}{Q}_{i}^{loss,h}{{-V}_{i}^{h}}^{2}({B}_{ij}+{B}_{sh})-{V}_{i}^{h}{V}_{j}^{h}{G}_{ij}\mathit{sin}\left({\theta }_{i}^{h}-{\theta }_{j}^{h}\right)\\ & \quad+ {V}_{i}^{h}{V}_{j}^{h}{B}_{ij}\mathit{cos}\left({\theta }_{i}^{h}-{\theta }_{j}^{h}\right)={Q}_{i}^{d,h}; \forall \left\{h\in H\right\}\end{aligned} $$

14 $${\left({{P}_{ij}}^{h}\right)}^{2}+{\left({{Q}_{ij}}^{h}\right)}^{2}\le {\left({ S}_{ij}^{max}\right)}^{2} ; \forall \left\{h\in H,i,j\in B\right\}$$

Different faults, such as single line-to-ground, double lines-to-ground, and line-to-line faults, are common in power networks. However, this study focuses specifically on the three-phase short-circuit fault, considered the most severe. The computation of this fault is detailed in Eq. (15). It is crucial that the fault current stays below the predefined threshold ${I}_{max}^{SC}$​, as specified in (16). ${\text{V}}_{i}^{h}(0)$ denotes the pre-fault voltage, and ${Z}_{ii}$ corresponds to the diagonal value of bus *i* in the impedance matrix.

15 $${I}_{i}^{SC}=\frac{{\text{V}}_{i}^{h}(0)}{{{Z}_{ii}}^{h}} ; \forall \left\{h\in H,i\in B \right\}$$

16 $${I}_{i}^{SC,h}\le {I}_{max}^{SC} ; \forall \left\{h\in H,i\in B \right\}$$

### TEP model

The TEP model under consideration seeks to economically identify and implement necessary upgrades to the electrical transmission system, ensuring it meets future electricity demand and accommodates EVs while maintaining reliable and efficient power delivery. The objective function, defined in (17), aims to minimize both investment (${C}^{inv}$) and operational (${C}^{op}$) costs associated with newly installed devices. Equation (18) represents the investment cost of newly installed transmission lines, generation units, ESSs, and var compensators. Meanwhile, (19) represents the operating cost of generation stations and ESSs.

17 $${OF}_{1}={C}^{inv}+{C}^{op}$$

18 $$ \begin{aligned}{C}^{inv}& =\sum_{\forall h\in H}{ F}_{CRF}\left(\sum_{\forall i,j\in B} {C}_{ij} ({N}_{L,ij}^{h}-{N}_{L,ij}^{h-1})+\sum_{\forall g\in G} \left({ C}_{G}^{invs} { \Delta P}_{G,g}^{h}+{ C}_{G}^{CCSS} {CCSS}^{CO2} { P}_{G,g}^{h}\right)+ \sum_{\forall r\in RES} { C}_{RES}^{invs} { \Delta P}_{RES,r}^{h}\right. \\ & \quad  \left. + \sum_{\forall b\in BAT} \left({c}_{sc,b}{ \Delta E}_{ESS,b}^{ h}+{c}_{pc, b} {\Delta P}_{ESS,b}^{h} +{c}_{pb, b} {\Delta P}_{ESS,b}^{h}+{B}_{rep, b} {N}_{rep, b}{ \Delta E}_{ESS, b}^{ h}\sum_{\forall \gamma \in {\Omega }_{Rep}}\frac{1}{{\left(1+\lambda \right)}^{{\gamma }_{b}\times {A}_{b}}}+{B}_{el, b} {\Delta P}_{ESS, b}^{h}\right)\right. \\ & \quad  \left. +\sum_{\forall sv\in VAR} \left({C}_{1}^{SVC}{\left({Q}_{SVC,s}^{h}-{Q}_{SVC,s}^{h-1}\right)}^{2}+{C}_{2}^{SVC} \left({Q}_{SVC,s}^{h}-{Q}_{SVC,s}^{h-1}\right)+{C}_{3}^{SVC}\right)+\sum_{\forall i,j\in B}{ C}_{ij}^{FCL} {X}_{FCL,ij}^{h}\right)\end{aligned} $$

19 $$ \begin{aligned}{C}^{op}& =\sum_{\forall h\in H} {F}_{CRF}\sum_{\forall g\in G}{ C}_{g}^{vop} {P}_{G,g}^{ h}+ { C}_{G,g}^{fop} { \Delta P}_{G,g}^{h}\\ &\quad+\sum_{\forall b\in BAT}\left({c}_{fixed, b} {\Delta P}_{ESS, b}^{h}\sum_{\forall y\in Y}\frac{{\left(1+{V}_{fc, b}\right)}^{y}}{{\left(1+\lambda \right)}^{y}}+\frac{{B}_{elec, b} \Delta T { P}_{ESS,b}^{h}}{ {\eta }_{ESS, b}}\sum_{\forall y\in Y}\frac{{\left(1+{V}_{elec, b}\right)}^{y}}{{\left(1+\lambda \right)}^{y}}\right)\end{aligned} $$

The capital recovery factor, denoted as ${F}_{CRF}$​, is determined by the formula $\frac{\lambda {\left(1+\lambda \right)}^{Y} }{{\left(1+\lambda \right)}^{Y}-1}$​, where *Y* is the project’s lifespan in years, and λ is the discount rate. The terms ${N}_{L,ij}^{h}$ and ${N}_{L,ij}^{h-1}$​ represent the number of circuits calculated for the route from node *i* to node *j* under scenarios *h* and *h*-1, respectively. Simultaneously, ${C}_{ij}$ denotes the investment cost per circuit for the *i*-*j* route in a million USD/circuit.

The second term in (18) pertains to the investment cost associated with new conventional generation units and carbon capture and storage systems (CCSSs)^51^. ${P}_{G,g}^{h}$ and ${\Delta P}_{G,g}^{h}$​ represent the total power generated by non-renewable units and its incremental value compared to the previous scenario. Meanwhile, ${C}_{G}^{invs}$​ is the investment cost coefficient of the generation station in USD/MW. ${CCSS}^{C{O}_{2}}$ denotes a factor estimating the CO_2_ released per MWh in kg/MWh. The parameter ${C}_{G}^{CCSS}$​ represents the cost of CCSS in million USD per kilogram of captured CO_2_. The third term in (18) covers the investment cost of RES. The fourth term formulates the investment cost of the installed ESS^49^. It considers various cost components, including the cost of the storage container (${c}_{sc,b}$​) in USD/MWh, the cost of the power conversion component (${c}_{pc, b}$) in USD/MW, and the ESS’s balance cost (${c}_{pb, b}$​) in USD/MW. Additionally, it incorporates the replacement cost of components (${B}_{rep, b}$​) in USD/MWh that may require replacement during the project’s lifespan and the end-of-life cost (${B}_{el, b}$​) in USD/MWh incurred due to ESS recycling. The variable ${A}_{b}$​ denotes the time until the ESS needs replacement.

The fifth term in (18) represents the investment cost of SVCs, with cost coefficients denoted by ${C}_{1}^{SVC}$, ${C}_{2}^{SVC}$ and ${C}_{3}^{SVC}$. The sixth term signifies the investment cost of the installed FCLs to preserve the short-circuit current within the predefined values.

In (19), the first term encompasses the fixed and variable operating costs associated with the generation units, while the subsequent term signifies the variable and fixed operating costs related to the ESS^49^. The factors denoting the variable and fixed operating costs of the generation units (expressed in million USD/MW) are denoted as ${C}_{g}^{vop}$ and ${C}_{G,g}^{fop}$ respectively. The fixed operating cost factor for the energy storage system is denoted by ${c}_{fixed, b}$ in million USD/MW, and the market energy price is represented by ${B}_{elec, b}$ in million USD/MWh. The rates of change for the fixed and market energy price costs of the energy storage system are respectively given by ${V}_{fc, b}$ and ${V}_{elec, b}$​. The efficiency of the energy storage system is denoted by ${\eta }_{ESS, b}$​. The TEP constraints are the number of circuits and size of FCLs, per each transmission line, is constrained by (20) and (21), while the constraints on reactive sources, which regulate the installed capacity, are explained in (22)^52^. $VAR$ is a set comprises candidate locations of SVCs.

20 $$\underset{\_}{{N}_{L,ij}^{h-1}}\le {N}_{L,ij}^{h}\le \overline{{N }_{L,ij}^{h}} ; \forall \left\{h\in H, i,j\in B\right\}$$

21 $$\underset{\_}{{X}_{FCL,ij}^{h-1}}\le {X}_{FCL,ij}^{h}\le \overline{{X }_{FCL,ij}^{h}} ; \forall \left\{h\in H, i,j\in B\right\}$$

22 $$\underset{\_}{{Q}_{SVC,s}^{h}}\le {Q}_{SVC,s}^{h}\le \overline{{Q}_{SVC,s}^{h} }; \forall \left\{h\in H,s\in VAR\right\}$$

From (23) to (27), the technical limitations of ESSs are delineated. In an ESS with a bidirectional converter (BDC), the charge and discharging of active power are governed by (23) and (24). In contrast, the quantity of injected or absorbed reactive power is determined by (25) and (26). By ensuring that the combined reactive powers absorbed and injected through BDCs do not surpass the maximal charged and discharged power capacity of the ESSs, inequality constraints (27) are implemented^53^. $BAT$ signifies the set of ESS’s candidate buses.

23 $$0\le {P}_{ESS,b}^{dch,h}\le \overline{{P}_{ESS,b}^{dch,h}} ; \forall \left\{h\in H,b\in BAT\right\}$$

24 $$0\le {P}_{ESS,b}^{ch,h}\le \overline{{P}_{ESS,b}^{ch,h}}; \forall \left\{h\in H,b\in BAT\right\}$$

25 $$0\le {Q}_{ESS,b}^{dch,h}\le \overline{{Q}_{ESS,b}^{dch,h}}; \forall \left\{h\in H,b\in BAT\right\}$$

26 $$0\le {Q}_{ESS,b}^{ch,h}\le \overline{{Q}_{ESS,b}^{ch,h}}; \forall \left\{h\in H,b\in BAT\right\}$$

27 $$\sqrt{{\left({P}_{ESS,b}^{h}\right)}^{2}+{\left({Q}_{ESS,b}^{h}\right)}^{2}}\le {S}_{b}^{ESS,K,max} ; \forall \left\{h\in \text{H},b\in BAT\right\}$$

${P}_{ESS,b}^{dch,h}$ and ${P}_{ESS,b}^{ch,h}$ are managed in each scenario based on the SOC of ESSs. ${P}_{ESS,b}^{dch,h}$ is calculated using (28), while ${P}_{ESS,b}^{ch,h}$ is obtained by (29). It relies on the rated power and the current SOC of the ESS selected.

28 $${P}_{ESS,b}^{dch,h} =\mathit{min}\left({P}^{ESS,b },\frac{{SOC}_{ESS,\text{b}}^{h}-{E}_{ESS,Kb}^{min}}{\Delta h}\right) ; \forall \left\{h\in \text{H},b\in BAT\right\}$$

29 $${P}_{ESS,b}^{ch,h} =min\left({P}^{ESS,b },\frac{{E}_{ESS,b}^{max}-{SOC}_{ESS,,b}^{h}}{\Delta h}\right); \forall \left\{h\in \text{H},b\in BAT\right\}$$

where SOC of the ESS built is defined using (30).

30 $$ SOC_{{ESS,{\text{b}}}}^{h}  = SOC_{{ESS,{\text{b}}}}^{{h - 1}}  + \eta _{{ESS,b}}^{{ch}} P_{{ESS,b}}^{{ch,h}}  - {\raise0.7ex\hbox{${P_{{ESS,b}}^{{dch,h}} }$} \!\mathord{\left/ {\vphantom {{P_{{ESS,b}}^{{dch,h}} } {\eta _{{ESS,b}}^{{dch}} }}}\right.\kern-\nulldelimiterspace} \!\lower0.7ex\hbox{${\eta _{{ESS,b}}^{{dch}} }$}}\forall \left\{ {h \in {\text{H}},b \in BAT} \right\} $$

## Optimization algorithm

To address the proposed problem effectively, the model is systematically segmented into two distinct layers. In the initial stage, the COA is strategically applied to the DR model, explained in (1) to (4). The primary aim of employing the COA is to ascertain the maximum level of load shifting that the network can feasibly accommodate. Subsequently, the HC model, outlined in (5) to (16), and the TEP model, outlined in (17) to (30), are simultaneously addressed.

Scaling metaheuristic algorithms to huge problem sizes or datasets may be computationally intensive and impractical, particularly for non-linear optimization models. By combining different algorithms, a hybrid approach can leverage the diverse search strategies of each component algorithm to explore the search space more comprehensively. This work applies the hybrid of PSO and COA to solve the HC and TEP models simultaneously. The PSO algorithm is employed to solve the HC model, while the COA is deployed to solve the TEP model. This composed approach tends to be more robust against the risk of premature convergence to suboptimal solutions.

### Crayfish optimization algorithm (COA)

The COA, initially presented by Jia et al.^54^ in 2023, draws its inspiration from the nuanced behaviors exhibited by crayfish during foraging, summer vacation, and competitive interactions^54^. These behaviors, integral to the algorithm, unfold across three distinctive stages — namely the summer resort stage, competition stage, and foraging stage — strategically designed to balance the algorithm’s exploration and exploitation aspects. The COA prioritizes exploration during the summer resort stage, while the subsequent competition and foraging stages emphasize exploitation. The algorithm’s temperature serves as a critical regulator governing the equilibrium between exploration and exploitation.

Crayfish participate in exploratory activities during the summer resort stage, which is marked by higher temperatures. Some of these behaviors include fighting for the same shelter or looking for shelter in caves. On the other hand, at ideal temperatures, crayfish display a variety of foraging behaviors depending on the amount of food that is available. Their food intake, in turn, affects the quantity of food ingested. Temperature modulation introduces more randomness into the COA and increases its global optimization potential when integrated into the exploration and exploitation processes.

The operational framework of the COA is expressed mathematically as follows^54^:

- **Step 1:** Define the number of iterations *T*_*max*,_ population size *N*, dimension *d*, upper bound *U*, and lower bound *B*. The initial population *X* is randomly generated based on the specified upper and lower bounds using (31).
  31 $$X=B+\left(U-B\right). rand$$
- **Step 2:** Determine the ambient temperature of the crayfish (*temp*) using (32) to decide the stage of the COA.
  32 $$temp=rand\times 15+20$$
  1. If *temp* > 30 and *rand* < 0.5, the COA enters the summer resort stage. In this stage, the COA generates a new position using the cave position (*X*_*shade*_) and crayfish position (*X*_*i*_^*t*^) according to the updated formula in (33). *X*_*shade*_ equals ${X}_{G}+{X }_{L}/2$. ${X}_{G}$ is the so-far-obtained optimal position, and ${X}_{L}$ denotes the best position of the current population. *C*_1_ is a constant that decreases with iterations.
    33 $${X}_{i}^{t+1}={X}_{i}^{t}+{C}_{1} \times rand\times \left({X}_{shade}- {X}_{i}^{t}\right)$$
  2. The COA enters the competition stage if *temp* > 30 and *rand* ≥ 0.5. During this stage, two crayfish compete for the cave based on (34) and obtain a new position using the cave position (*X*_*shade*_) and the positions (${X}_{i}^{t}$) of the two crayfish. ${X}_{r}^{t}$ is a random crayfish selected from the current population.
    34 $${X}_{i}^{t+1}={X}_{i}^{t}+{X}_{shade}- {X}_{r}^{t}$$
  3. If *temp* ≤ 30, the COA enters the foraging stage. The food intake *p* and food size *Q* determined using (35) and (36), respectively.
    35 $$p={C}_{2}\times \left(\frac{1}{\sqrt{2\times \pi }\times \sigma }exp\left(\frac{{\left(temp-\mu \right)}^{2}}{2{\sigma }^{2}}\right)\right)$$
    36 $$Q={C}_{3}\times rand\times \frac{{F}_{i}^{t}}{{F}_{food}^{t}}$$
    where ${C}_{3}$ is a constant and equal 3. ${F}_{i}^{t}$ and ${F}_{food}^{t}$ are the fitness values of ${X}_{i}^{t}$ and the food location, respectively.
    If *Q* > (*C*_3_ + 1)*/*2, the food is shredded according to (37). Then, calculate the new position using (38).
      37 $${X}_{food}=exp\left(\frac{-1}{Q}\right)\times {X}_{food}$$
      38 $${X}_{i}^{t+1}={X}_{i}^{t}+{X}_{food}\times p\times \left(cos\left(n\times \pi \times rand\right)-cos\left(n\times \pi \times rand\right)\right)$$
    If *Q* ≤ (*C*_3_ + 1)*/*2, calculate the new position using (39).
      39 $${X}_{i}^{t+1}=\left({X}_{i}^{t}-{X}_{food}\right)\times p+p\times rand\times {X}_{i}^{t}$$
- **Step 3:** Evaluate the population and determine whether to exit the cycle. If the termination condition is not met, return to step 2.

### Particle swarm optimizer (PSO)

PSO is known due to its simplicity and ease of implementation. It has been successfully applied to various optimization problems, including function optimization, TEP, DR optimization problems, and HC planning models. However, like many optimization algorithms, its performance relies on the problem being solved and the choice of parameters. Like any meta-heuristic algorithm, PSO starts by generating random solutions, and the candidate positions of individuals are updated throughout a predefined number of iterations using (37) and (38). *w* is the inertia weight, while *C*_1​_ and *C*_2_​ are acceleration coefficients^55^.

40 $${X}_{i}^{t+1}={X}_{i}^{t}+{v}_{i}^{t+1}$$

41 $${v}_{i}^{t+1}=\text{w }{v}_{i}^{t}+{C}_{1}\left({X}_{L}-{X}_{i}^{t}\right)+{C}_{2}\left({X}_{G}-{X}_{i}^{t}\right)$$

### Proposed hierarchy-solving strategy based on PSO-COA

**Figure 4:**
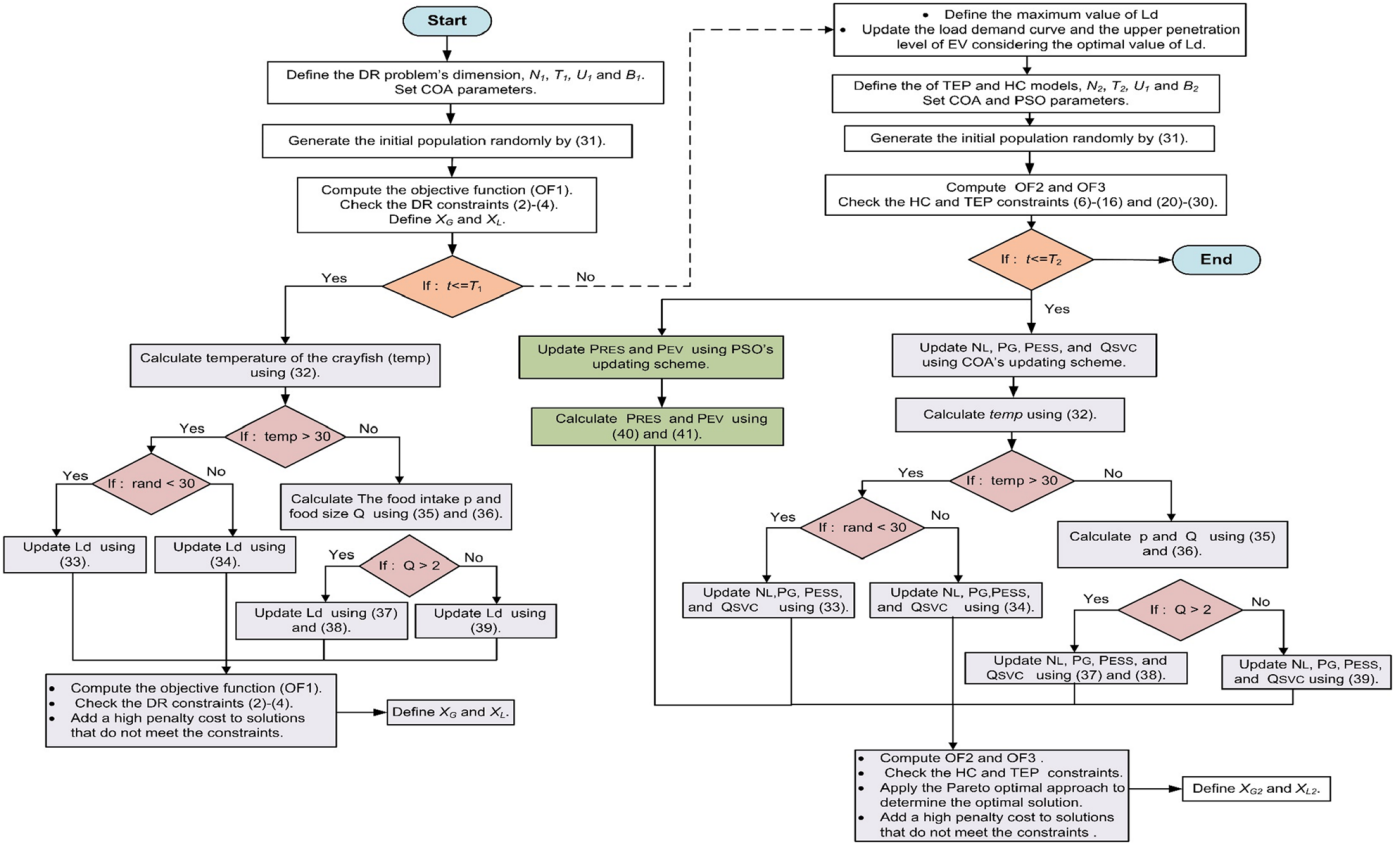
Flowchart of the proposed hierarchy algorithm.

Figure 4 explains the intricate structure of the suggested hierarchical algorithm. It is intended to function on two different layers. The first layer intentionally uses the COA to address the DR model, making it easier to estimate the allowable amount of load shifting that falls under the authority of system operators. This layer’s optimization problem aims to maximize the load-shifting capacity in a single-objective manner. Meanwhile, the second layer handles the HC and TEP models, which treats them as components of a multi-objective optimization problem. In this layer, the COA and the PSO algorithm are harnessed to navigate the solution space and synergistically optimize both the HC and TEP models. This hierarchical approach ensures a comprehensive resolution, optimizing load-shifting capabilities in the first layer and balancing the trade-offs between HC and TEP objectives in the second layer. Within the initial layer, the COA algorithm is initialized with meticulous consideration of the upper (*U*_1_) and lower (*B*_1_) bounds encapsulating the load shifting levels, as expounded in (31). The problem dimension and termination criteria (*T*_1_) are distinctly defined, providing a robust foundation for the algorithm's execution. Throughout each iteration, the COA dynamically computes a new solution for the *L*_*d*_ by leveraging its updating scheme, precisely detailed in (32)–(39). The iterative process continues until the predefined maximum number of iterations, *T*_1_, is reached. Upon reaching this threshold, the algorithm concludes its execution, and the optimal value for *L*_*d*_ is conclusively determined. Upon determining the optimal value of *L*_*d*_, the load demand curve and the maximum EV penetration level undergo updates, reflecting the shift in load distribution across hours. Subsequently, the second layer of the algorithm is engaged to solve a two-level cascading problem. The first level involves the refinement of decision-making variables for the HC model (${P}_{RES,r}^{h}$ and ${P}_{EV,e}^{h}$), while the second level focuses on updating variables for the TEP model ${{N}_{L,ij}}^{h}$, ${P}_{G,g}^{h},$ ${P}_{ESS,b}^{h}$ and ${Q}_{SVC,s}^{h}$). Algorithm 2 presents this process by initializing a population of size *N*_2_ using defined upper (*U*_2_) and lower (*B*_2_) bounds for HC and TEP variables. Iterations persist until the termination criteria of the second layer (*T*_2_) are met. New solutions are iteratively computed using PSO for HC parameters, and COA for TEP parameters, with the objectives (OF_2_ and OF_3_) recalculated at the conclusion of each iteration. This systematic approach ensures a comprehensive and iterative optimization process for both HC and TEP models within the hierarchical algorithm.

This study adopts the Pareto optimal approach to determine the optimal solution. A criterion for dominance is established to compare two or more objectives, defined as follows: Solution *X*_1_ dominates solution *X*_2_ if:

For all objectives *o* in the set {*1, 2, …, k}*:

42 $${\forall }_{o}\in \left\{\text{1,2},\dots \dots k\right\}: {f}_{o}\left({X}_{1}\right)\le {f}_{o}\left({X}_{1}\right)$$

43 $${\exists }_{o}\in \left\{\text{1,2},\dots \dots k\right\}: {f}_{o}\left({X}_{1}\right)< {f}_{o}\left({X}_{1}\right)$$

During each iteration, the non-dominated solutions are stored in a repository. The identified solution space is recorded, and the optimal memory location of individuals is updated. Any individuals in the current population that are dominated by others are included in the repository, thereby increasing its size. The repository’s members are then re-evaluated, and any dominant members are removed to reduce the repository’s population size. If the number of individuals exceeds the maximum capacity, a portion of the population is removed, and the tabulation process restarts.

## Results and discussion

The investigation was conducted utilizing two benchmark systems: Garver network and the IEEE 24-bus system. Simulations were executed using MATLAB r2021a software, ensuring a robust and reliable analytical framework. A total of twenty distinct simulation runs were performed to yield comprehensive insights, enhancing the statistical robustness of the findings. The Garver network, comprising six buses, three generation units, and five load buses, served as a benchmark for assessing the impact of the proposed strategies. In this network, buses 2 and 5 emerged as promising locations for the integration of new wind units, while buses 2, 5, and 6 were kept as potential sites for the deployment of ESSs and SVCs. The system parameters are detailed in^56^. The IEEE 24-bus system, a more extensive power network, involves 24 buses, 11 generation units, and 17 load buses. Wind unit installation potential was identified at buses 3, 10, and 19, while ESSs and SVCs were considered for deployment at buses 2, 6, 8, 14, 20, 21, 22, and 24. System data for this network is available in^57^. Both systems incorporated compressed air energy storage systems (CAESS), with characteristic details outlined in^58^. The cost information for generation units and CCSSs is provided in^51,59^.

### Impact of the load curve behavior on the level of DR

**Figure 5:**
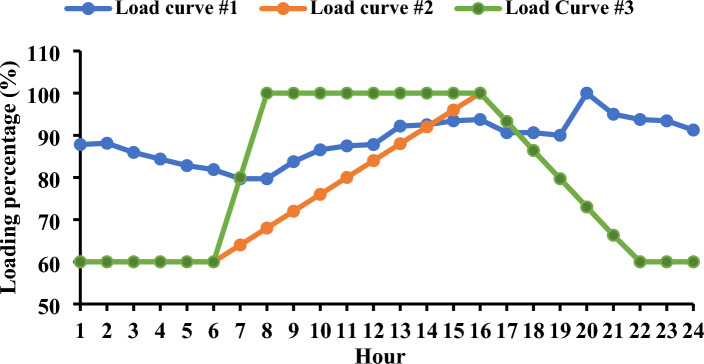
Adopted load curves used in this study.

Figure 5 illustrates the three load curves employed in this study^60^. The results indicate a substantial correlation between the behavior of load demand in power systems and the system’s capacity for accommodating load shifting at various hours.

**Figure 6:**
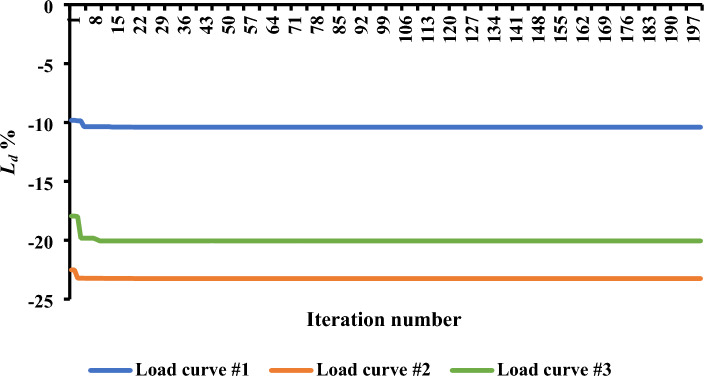
Convergence curves for the estimated load shifting levels.

**Figure 7:**
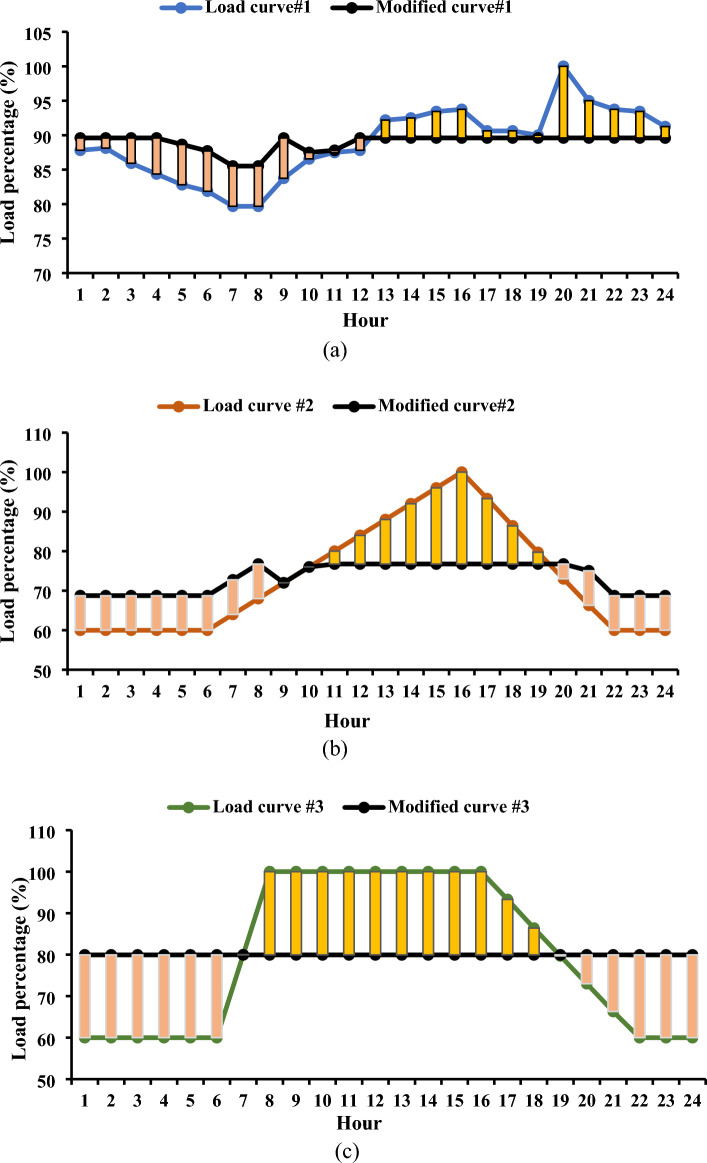
Modified load curves resulting from the use of DR.

Figure 6 provides a graphical representation of the maximum levels of load shifting observed, revealing values of 10.4%, 23.25%, and 20.05% for the first, second, and third load curves, respectively. These variations in load shifting are intricately linked to the energy demands throughout the day. For instance, in the first load curve in the Garver network, the total load energy reached 16.2 GWh/day, while the second and third load curves registered 13.36 and 14.58 GWh/day, respectively. The energy demands in the IEEE 24-bus system were 60.77, 50.12, and 54.68 GWh/day for the three curves, respectively. The observed trend indicates that, as energy requirements escalate, the level of controlled load diminishes, potentially impacting the efficacy of DR programs. Figure 7 offers a detailed depiction of the load curve alterations resulting from the implementation of *L*_*d*_. In the first curve, the load demand fluctuates between 85.53 and 89.6% throughout the day.

Conversely, a noticeable reduction in the peak load is evident for the second and third load curves, remaining below 76.75% and 79.95%, respectively, of the peak load observed in the base curves. These minor variations highlight the complex dynamics at work and the possible efficiency gains attained by employing innovative load management techniques, offering insightful information for enhancing the performance of the power system. The results saved from both the Garver network and the IEEE 24-bus system elucidate a noteworthy observation: irrespective of the varying amounts of peak load, the percentage of load shifting remains consistent for similar load behaviors. This uniformity is underscored by the finding that, for both systems, the maximum *L*_*d*_ achieved 10.4% of the respective peak load, as observed in the case of the first load curve.

### Impact of the DR on the TEP

#### Garver network

**Figure 8:**
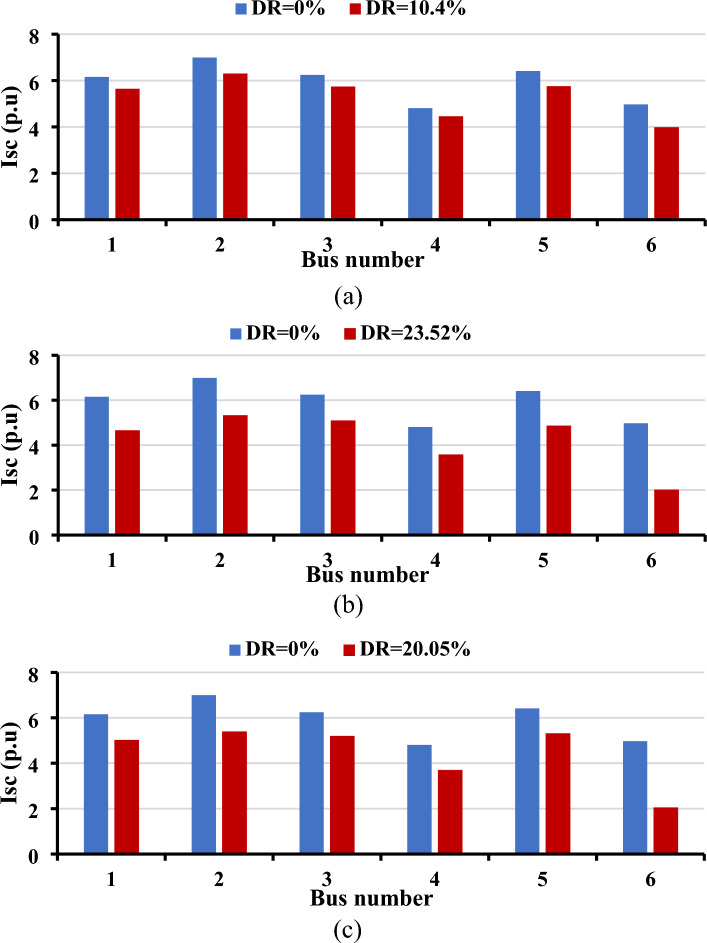
Impact of DR on the short-circuit currents for the Garver network: (**a**) load curve#1, (**b**) load curve#2, and (**c**) load curve#3.

Figure 8 illustrates the short-circuit currents at each bus resulting from the presence or absence of DR. All simulations were conducted with the constraint that the short-circuit current should not exceed seven p.u. The findings underscore the effectiveness of strategically optimizing the location and size of new circuits in managing short-circuit currents without the need for FCLs. Across all three load curves, the implementation of DR played a pivotal role in reducing short-circuit currents. Moreover, the adoption of DR contributed to a noteworthy decrease in the required capacity of SVCs and the number of newly installed circuits, as detailed in Table 2. Specifically, a reduction of at least two circuits and 66.29 MVAR was observed due to the implementation of DR for curve#1, 180.6 MVAR and 132.5 MVAR for Curve#2 and Curve#3; respectively.

**Table 2:** Installed projects required for enhancing the usage of EVs.

Garver system							
Case study	Added circuits	Added FCLs (p.u)			Added SVCs (MVAR)		
		Curve#1	Curve#2	Curve#3	Curve#1	Curve#2	Curve#3
Without DR (FCL)	1-5(1),2-3(2), 3-5 (1), 2-6(2), 4-6(1)	0	0	0	328.7	328.7	328.7
With DR	1-5(1)*, 2-3(2), 2-6(1)*, 4-6(1)	0	0	0	262.41	148.1	196.2

IEEE 24-bus system							
Case study	Added circuits	Added FCLs (p.u)			Added SVCs (MVAR)		
		Curve#1	Curve#2	Curve#3	Curve#1	Curve#2	Curve#3
Without DR	1-2(1), 7-2 (1), 7-8(1)	0	0	0	747.54	747.54	747.54
Without DR (FCL)		2.93 + i0.48	2.93 + i0.48	2.93 + i0.48	747.54	747.54	747.54
With DR		0	0	0	655.47	390.15	526.4

*Indicates new circuits added for Load curve#1 only.

#### The IEEE 24-bus system

**Figure 9:**
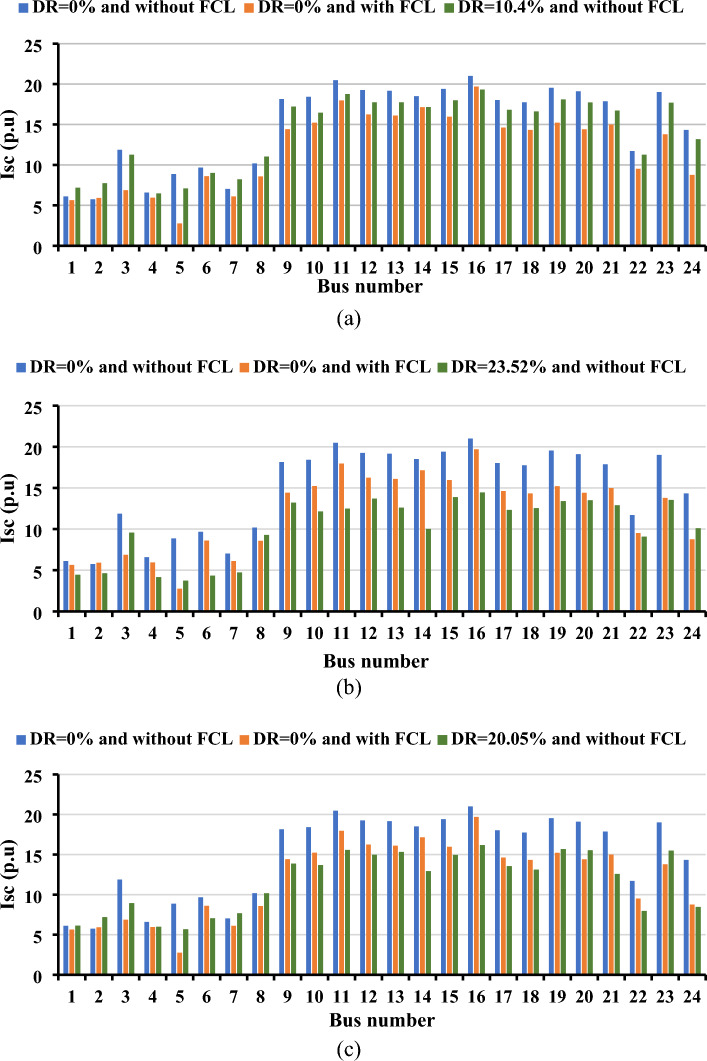
Impact of DR on the short-circuit currents for the IEEE 24-bus system: (**a**) Load Curve#1, (**b**) Load Curve#2, and (**c**) Load Curve#3.

The impact of DR on TEP is explored in Fig. 9. All simulations were conducted with strict adherence to the maximum permissible short-circuit current level, set at 20 p.u. The results from the three curves exhibit a notable reduction in short-circuit current compared to scenarios where a demand response strategy is not employed. In the baseline case without DR, FCLs are necessary to keep the short-circuit current within acceptable limits, as detailed in Table 2. The findings also indicate a significant decrease in the required SVC capacity, ranging between 92.07 MVAR and 357.39 MVAR, with the implementation of DR. Despite the uniformity of the system across all curves, the variance in load behavior throughout the day influences the SVC requirements. Importantly, the results show a consistent count of installed circuits across all cases for the 24-bus system.

### Impact of the TEP and DR on the HC of EVs

#### Garver network

**Figure 10:**
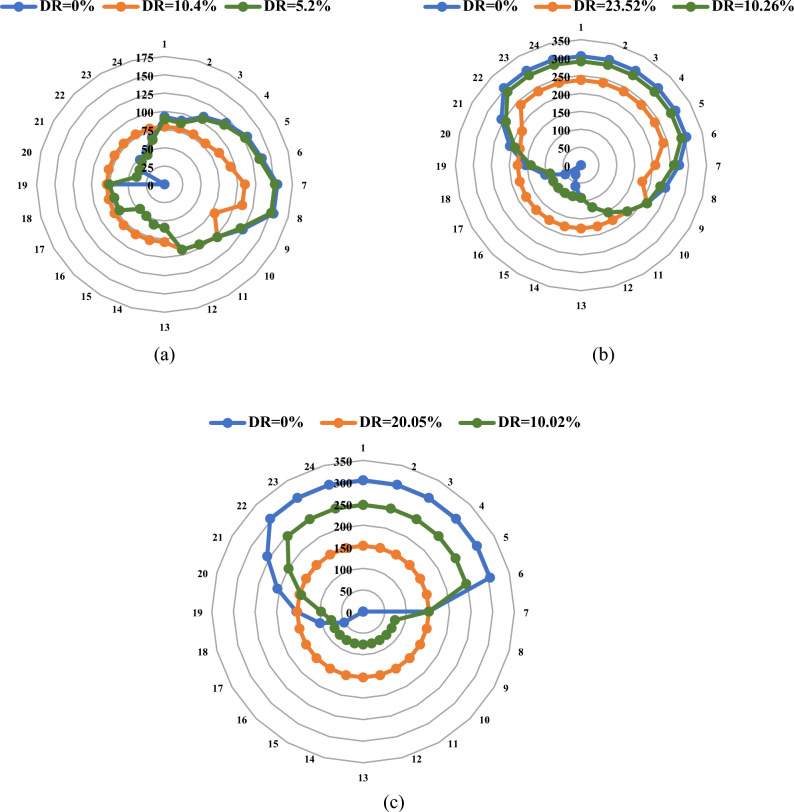
EV penetration in MW for the Garver system: (**a**) Load Curve#1, (**b**) Load Curve#2, and (**c**) Load Curve#3.

Figure 10 visually depicts the fluctuations in HC levels of EVs across various case studies. It is crucial to emphasize that these case studies were conducted, accounting for the total peak loads in each hour, with a stringent limit ensuring that, even in the presence of EVs, the aggregate load does not surpass 760 MW.

The findings emphasize the efficacy of DR in governing the penetration level of EVs throughout the day. Notably, the total MWh of EV charging over the day, with and without DR, across the three cases, remains consistent. However, a careful selection of the appropriate DR level enables a discernible increase in the penetration level of EVs during peak hours, as demonstrated in Fig. 10. This enhancement is a challenging feat to achieve without the strategic implementation of DR. The introduction of EV charging stations into the power system induces a substantial surge in peak demand, precipitating overloading on transmission lines and generation stations.

In Load Curve#1, the peak of demand occurs at hour 20, rendering it operationally unfeasible to accommodate EV charging without straining the system. The strategic application of a DR measure at a rate of 10.4% not only facilitated the elevation of EV penetration during hour 20 but also demonstrated an extended impact over a broader timeframe, spanning from hour 13 to hour 24, as shown in Fig. 10a. Such a sustained enhancement proves challenging in the absence of DR involvement. Implementing a DR measure of 5.2% resulted in the HC curve of EV reflecting that of the base case, yet with a pivotal shift—it became feasible to authorize EV charging even during the peak hour at hour 20. Notably, the HC of EVs over the entire day, whether with or without DR, equated to approximately 2.03 GWh per day. This finding underscores the resilience and adaptability of the proposed DR strategies in reshaping the temporal dynamics of EV charging, crucially expanding opportunities for integration during peak demand periods and ensuring sustained operational efficiency.

In the adapted Load Curve#2, the peak load remains below 583.3 MW, providing an expanded window for EVs to charge throughout the day. Remarkably, the total HC reached approximately 4.87 GWh, a consistent value whether employing DR strategies or not. This result emphasizes the pivotal role of load curve modifications, showcasing that variations in load center behavior significantly influence HC levels, as shown in Fig. 10b. Notably, Load Curve #2 exhibited a 2.85 GWh increase compared to Load Curve#1, underscoring the tangible impact of load center adjustments on the system’s overall capacity to host EV charging. The influence of DR was particularly conspicuous in redistributing the EV load from low-demand hours, thus enabling a more significant number of EVs to charge during peak load periods, as observed in the original curve. DR, as demonstrated, played an effective role in regulating the charging behavior of EVs. This facilitated a more careful distribution of EV charging across different demand periods and empowered the system operator with the flexibility to select the optimal charging behavior for EVs, aligning with operational efficiency and grid reliability considerations.

Within the dynamics of Load Curve#3, an expanded peak load period spanning approximately nine hours poses challenges for EV charging. In response, applying a DR mechanism at a robust 20.05% effectively ameliorated this issue by arranging an equitable distribution of EV charging periods throughout the day, as visually represented in Fig. 10c. Nevertheless, recognizing the necessity for alignment with realistic charging behavior, the DR level can be judiciously attenuated to a more suitable threshold, exemplified by a reduction from 20.05 to 10.02%. Crucially, it is noteworthy that the HC of EVs in this scenario reached approximately 3.65 GWh per day. This finding underscores the pivotal role of DR in modulating EV charging dynamics, providing a versatile solution to the challenges posed by prolonged peak load durations. The ability to fine-tune the DR level adds a layer of adaptability, ensuring compatibility with practical charging behaviors while maintaining an optimal balance in the hosting capacity of EVs throughout the day. This nuanced approach enhances the applicability of EV charging strategies in diverse operational scenarios, furthering the integration of electric vehicles into the broader energy landscape.

#### The IEEE 24-bus system

**Figure 11:**
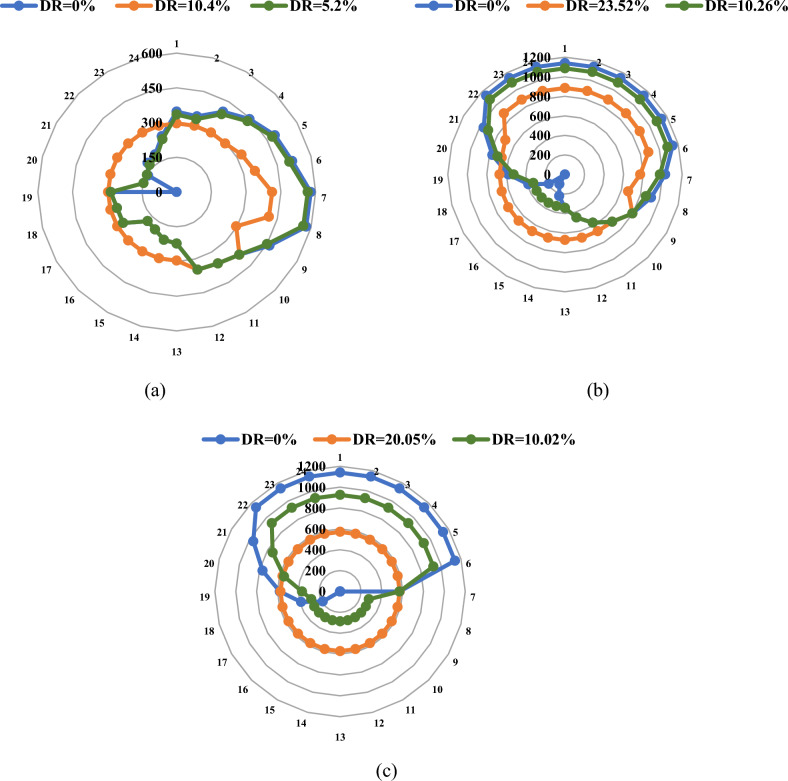
EV penetration (in MW) for the IEEE 24-bus system: (**a**) Load Curve #1, (**b**) Load Curve #2, and (**c**) Load Curve #3.

The findings obtained from the IEEE 24-bus system consistently validate and affirm the outcomes observed in the Garver network. Notably, the efficacy of DR in orchestrating the charging periods of EVs is pronounced, showcasing its robust regulatory impact. Interestingly, this regulatory effect does not significantly influence the maximum HC of EVs that can be seamlessly integrated into power networks. This observation is clearly illustrated in Fig. 11, where the charging behavior across all cases mirrors that of the Garver network when expressed as a percentage of peak load. Significantly, the uniformity in charging behavior across different systems underscores a crucial insight: the charging scenario is primarily shaped by the loading curve, and the behavior of loads emerges as the paramount factor in studying the hosting capacity of power networks. Consequently, regardless of the specific system under consideration, the obtained results indicate a common trend, suggesting that outcomes derived from one system’s load curve can be generalized and applied to other systems. This cross-system consistency enhances the broader applicability of insights gained from studying HC, facilitating a more comprehensive understanding of the dynamic interplay between load behaviors and EV charging scenarios in diverse power network configurations.

Upon evaluating the three load curves depicted in Fig. 5, discernible distinctions emerge, particularly in their impact on the penetration of EVs. Load Curve #1, in the optimal scenario, permits a lower penetration of EVs compared to the other two curves, with a maximum allowable DR not surpassing 10.4%. This constraint directly influences the achievable level of EV integration. The EV charging energy over the day varies, with Load Curve #1 reaching 7.62 gigawatt-hours per day and Load Curves #2 and #3 registering higher values at 18.27 GWh/day and 13.72 GWh/day, respectively. The implementation of DR distinctly demonstrates its efficacy in orchestrating a shift in EV load to off-peak hours. This strategic application of DR proves to be the most efficient approach, facilitating the charging of EVs during peak hours without imposing undue stress on the network’s components.

### Impact of the TEP and DR on the HC of RES

#### Garver network

**Figure 12:**
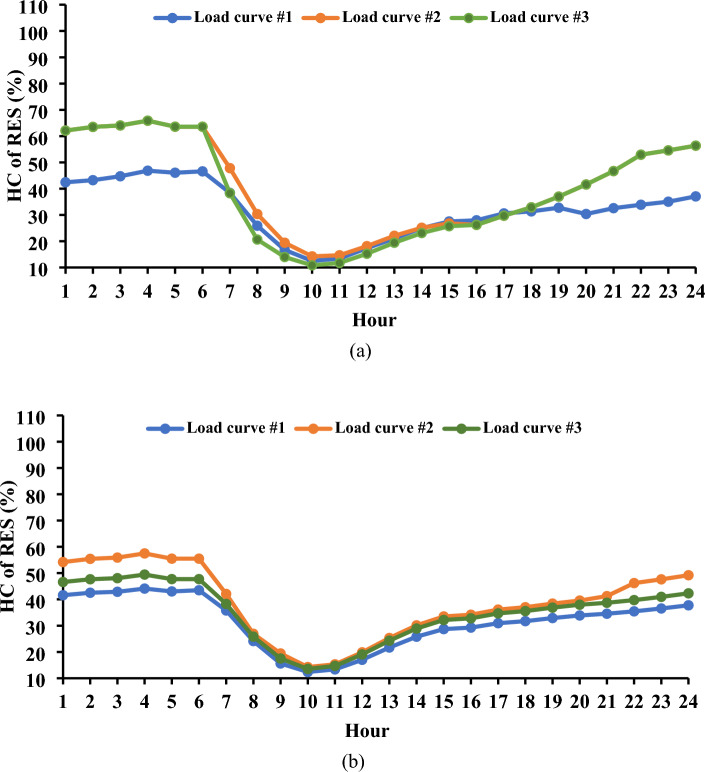
HC of RES for the Garver system: (**a**) without DR, and (**b**) with DR.

Figure 12 and Table 3 collectively depict the nuanced interplay of TEP and DR in influencing the HC of RES. The integration of RES into power networks introduces a transformative dynamic, necessitating a reassessment of the type and scale of projects required. The simulation outcomes underscore the indispensability of new circuits along designated routes to ensure a consistent power supply to the load, thereby augmenting the seamless integration of RES, particularly pronounced in the context of Load Curve #1.

**Table 3:** Installed projects required for enhancing the usage of RES.

Garver system							
Case study	Added circuits	Added FCLs (ohm)	Added SVCs (MVAr)	Added ESSs	Total cost (million USD)		
					Curve#1	Curve#2	Curve#3
Without DR	2–3 (2),3–5 (2), 4–6(2)	0	283.9	0	176.84	176.82	176.83
With DR	3–5 (2), 4–6(2)	0	234.5	0	166.53	155.11	158.83

IEEE 24-bus system							
Case study	Added circuits	Added FCLs (p.u)	Added SVCs (MVAr)	Added ESSs	Total cost (million USD)		
					Curve#1	Curve#2	Curve#3
Without DR	1-2(1), 7-2 (1), 7-8(1)	0.6130 + i0.09	590.8	0	492.83	492.75	492.9
With DR		0	0	0	492.83	468.49	468.51

The integration of DR emerges as a pivotal factor impacting the size of the requisite generation units, as detailed in Table 3. In case of Load Curve #2, the absence of a need for additional thermal units is notable, with the integration of RES proving sufficiently adept at meeting the load requirement. This outcome translates into a substantial decrease of 21.7 million USD in the investment cost compared to the base case. For Load Curve #3, the incorporation of the contemplated DR strategy emerges as a transformative force, markedly reducing the size prerequisites of the essential generation unit. A 20% reduction, as compared to the base case, attests to the benefits emanating from the careful implementation of the DR approach. These results not only highlight the substantial advantages of employing DR but also underscore its role in achieving a nuanced equilibrium, showcasing the intricate balance attainable through the synergistic integration of TEP and DR. This integrated approach not only optimizes the HC of RES but also furnishes valuable insights for enhancing the efficiency and cost-effectiveness of power network planning.

While the utilization of ESS proves indispensable for augmenting the integration of RES and extracting maximal benefits for power systems, it is noteworthy that ESSs currently do not enjoy economic viability comparable to conventional generation units. The proliferation of ESS within power networks becomes much more prevalent under circumstances with a substantial reduction in the output of generation units. In such instances, deploying ESS becomes imperative to safeguard system reliability, underscoring the critical role ESS plays in maintaining the resilience and stability of the overall power infrastructure.

The results of the simulations also show that the maximum penetration level of HC is negatively impacted by the addition of DR. Nonetheless, as Fig. 10 illustrates, a noticeable and advantageous impact is noted in raising the minimum amount of HC throughout the day. Upon the implementation of DR, the maximum HC percentage over the day witnessed a reduction from 46.84 to 44.1% for the first curve, from 65.87 to 55.49% for the second curve, and from 65.87 to 49.44% for the third curve. Adopting the load-shifting strategy engenders an increase in the load demand during specific hours compared to the base case, necessitating heightened dispatching from thermal generation units. Conversely, the strategic deferment of some loads during certain hours leads to a marginal decrease in the load demand in comparison to the standard case. This nuanced shift contributes to a slight improvement in the lower level of HC estimated over the day. The lower level of HC anticipated for the day shows a little improvement as a result of this slight shift. Figure 10 provides an obvious example of this improvement, as Load Curve #3 shows an improvement of 2.7%. These results highlight the complexities of DR deployment and its subtle consequences on minimum and maximum HC levels, providing important information for enhancing power network design techniques.

#### The IEEE 24-bus system

**Figure 13:**
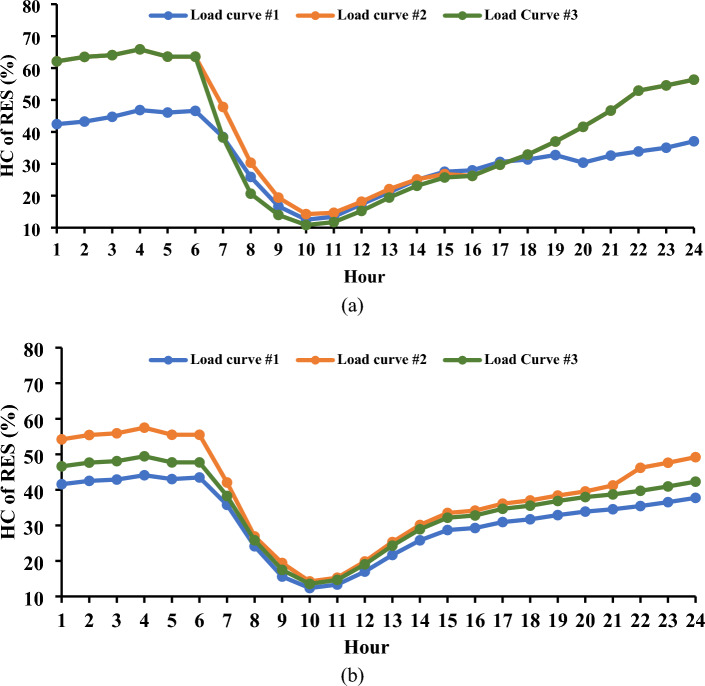
HC of RES for the IEEE 24-bus system: (**a**) without DR, and (**b**) with DR.

The enhanced system infrastructure details are outlined comprehensively in Table 3. Meanwhile, the HC levels over the 24 h are presented in Fig. 13. The insights derived from the IEEE 24-bus system consistently corroborate the conclusions drawn from the Garver network. The strategic implementation of the DR strategy emerges as a pivotal factor in augmenting the economic efficiency of the planned system. Notably, the second load curve exhibited a remarkable 23.52% reduction in the maximum peak load compared to the base case. Consequently, there was no need for additional non-RES, as the output from wind stations and existing generation units proved sufficient to meet the load requirements throughout the day. This reduction in dependency on conventional units not only contributed to economic gains but also yielded environmental benefits by curbing the amount of CO_2_ emissions. The findings underscore the dual advantages of DR implementation, showcasing economic feasibility and the potential for significant environmental impact, thus reinforcing the importance of strategic planning and integration of renewable resources in contemporary power systems.

### Efficiency of the proposed hybrid algorithm in solving the problem

**Figure 14:**
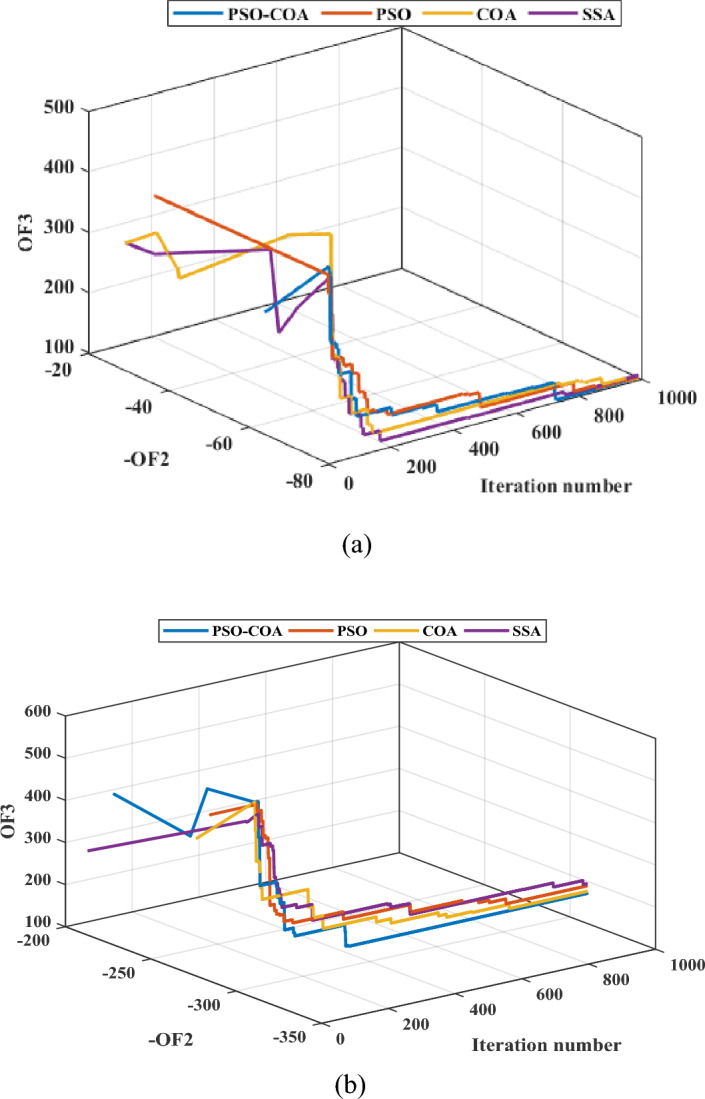
Convergence curves for the Garver network: (**a**) EV-HC with DR and TEP, and (**b**) RES- and EV-HC with DR and TEP.

**Figure 15:**
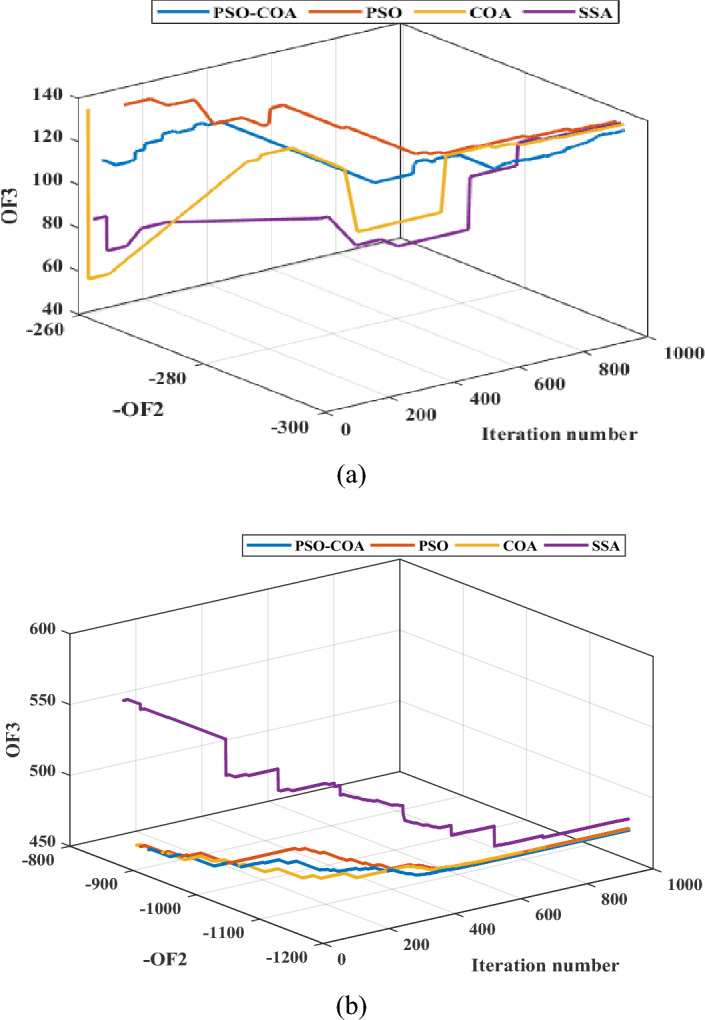
Convergence curves for the IEEE 24-bus system: (**a**) EV-HC with DR and TEP, and (**b**) RES- and EV-HC with DR and TEP.

The performance of the proposed PSO-COA is compared with that of PSO, COA, and the salp swarm optimizer (SSA)^61^. Simulation results for both the Garver network and the IEEE 24-bus system are presented in Tables 4 and 5, respectively. Additionally, the convergence curves for all algorithms are depicted in Figs. 14 and 15. The simulations were conducted for Load Curve #1, addressing scenario number 20, with results aggregated over 20 independent runs, each spanning 1000 iterations. The outcomes across both systems demonstrate the superior effectiveness of the proposed algorithm in obtaining high-quality solutions. The decomposition of the decision-making variables in the problem aids the proposed strategy by reducing the number of variables, thereby enhancing the algorithm’s ability to explore a broader range of search areas and improving the exploitation characteristics of both COA and PSO.

**Table 4:** Simulation results of PSO-COA, PSO, COA, and SSA for the Garver network.

Garver network				
Case study	Algorithm	Best (-OF2)	Best (OF3)	Time (s)
EV-HC with DR and TEP	PSO-COA	− 79.04	98.4	511.26
	PSO	79.04	99.84	405.02
	COA	79.04	98.7	497.12
	SSA	79.04	102.51	376.2
RES- and EV-HC with DR and TEP	PSO-COA	− 309.77	170.74	537.2
	PSO	− 309.77	180.70	421.1
	COA	− 309.77	175.74	523.4
	SSA	− 309.77	194.18	394.7

**Table 5:** Simulation results of PSO-COA, PSO, COA, and SSA for the IEEE 24-bus system.

The IEEE 24-bus system				
Case study	Algorithm	Best (-OF2)	Best (OF3)	Time (s)
EV-HC with DR and TEP	PSO-COA	− 296	131.45	1016.1
	PSO	− 294.7	134.01	905.3
	COA	− 296	134.01	944.7
	SSA	295.49	134.03	852.03
RES- and EV-HC with DR and TEP	PSO-COA	− 1161.7	470.68	1081.02
	PSO	− 1161.7	471.9	940.8
	COA	− 1161.7	471.83	1012.4
	SSA	− 1161.7	478.6	894.1

## Conclusions

This research proposed an enhanced HC model integrated with TEP and DR. This integration leverages the complementary strengths of DR and TEP to ascertain the maximal capacity of power networks for the seamless integration of RES and EVs. The model introduces the incorporation of SVCs, ESSs, and FCLs, offering technical and economic enhancements to HC strategies. A hierarchical optimization algorithm was introduced to address the complexities, breaking down the planning model into three levels for problem simplification and generating high-quality solutions.

In addition, a hybrid multi-objective optimization technique that combined the PSO and COA was presented to effectively solve the suggested model. This research opens up new possibilities for improved planning and integration strategies by advancing optimization approaches in this field. The results derived from the analysis of the Garver and the IEEE 24-bus test systems affirm several key insights. Firstly, the fluctuations in load shifting intricately correlate with the energy demands throughout the day. This observed pattern suggests that as energy requirements increase, the effectiveness of DR programs may be impacted, leading to a reduced controlled load. The maximum levels of load shifting observed were about 10.4%, 23.25%, and 20.05% for three load curves that varied in total load energy over the day.

Implementing DR is pivotal in reducing short-circuit currents across three load curves. For the Garver network, the maximum short-circuit current decreased by at least 0.7 p.u. compared to the case of ignoring the DR strategy, and by about 1.7 p.u. for the 24-bus system. Furthermore, adopting DR significantly decreases the required capacity of SVCs and the number of newly installed circuits. The use of DR led to a reduction of at least two circuits and 66.29 MVAR for the Garver network. In the 24-bus system, the size of FCL decreased by 2.97 per unit, and the size of SVCs decreased by about 92.1 MVAR.

The results emphasize the efficacy of DR in managing the penetration level of EVs throughout the day. Remarkably, the total MWh of EV charging remains consistent over the day across the three cases, whether DR is implemented or not. However, judicious selection of an appropriate DR level enables a noticeable increase in the penetration level of EVs during peak hours, a feat challenging to achieve without strategic DR implementation. The penetration level of EVs increased by about 176.7 MW for the Garver network and by about 670.3 MW for the 24-bus system in some case studies.

The integration of RES into power networks introduces a transformative dynamic, prompting a reevaluation of the type and scale of projects required. While ESSs are essential for enhancing RES integration and maximizing benefits for power systems, it is noteworthy that ESSs currently lack economic viability compared to conventional generation units. The simulations also reveal that the introduction of DR has an adverse impact on the maximum penetration level of RES HC. However, a discernible and beneficial effect is observed in enhancing the minimum level of HC over the course of the day. The minimum percentage of HC increased by 2.7% due to the use of the DR program.

The performance of the proposed PSO-COA is compared with that of PSO, COA, and SSA. The outcomes across both systems demonstrate the superior effectiveness of the proposed algorithm in obtaining high-quality solutions. The PSO-COA has the limitation of longer computation times compared to PSO, COA, and SSA. Specifically, the computation time increased by at least 2.77% for the Garver network and 2.55% for the 24-bus system.

Further research is essential to examine the effects of short-term uncertainty in EV availability and the impact of smart charging on battery degradation, aiming to enhance the accuracy of operational issues. Moreover, studies are required to assess various hybridization schemes of meta-heuristic algorithms, focusing on the quality of the solutions obtained.

## Data Availability

The datasets generated during the current study are not publicly available due to their large size but are available from the corresponding author on reasonable request.
